# Reply

**DOI:** 10.1002/acr2.11476

**Published:** 2022-06-21

**Authors:** Banafsheh Ghavidel‐Parsa, Ali Bidari

**Affiliations:** ^1^ Razi Hospital and School of Medicine Guilan University of Medical Sciences Rasht Iran; ^2^ Iran University of Medical Sciences Tehran Iran

*To the Editor*:

We thank the American College of Rheumatology (ACR) *Open Rheumatology* journal for the opportunity to respond to the letter by Wolfe et al (1) about our recently published original article. In this study, we developed and validated a new tool for the diagnosis of fibromyalgia (FM), Nociplastic‐Based Fibromyalgia Features (NFF). We would like to mention some important points in response to the authors.

Wolfe et al (1) were concerned with the method of our diagnosis used in the NFF study. They mentioned that this method was probably biased and idiosyncratic and identified different patients with less severity than is usual among patients with rheumatology diagnoses (1). Because there is no gold standard for FM diagnosis, the definition of FM is always arguable and open to question (2). The reference standard in the NFF study, like in previous studies, was expert diagnosis, which seems to be the best strategy for the diagnosis of FM until now. Expert clinical judgment entails the time spent in physical examination, evaluation, and interpretation of each individual symptom and psychosocial factors, which cannot be assessed by any criteria (3). Therefore, on exploring biomarkers for FM diagnosis, the diagnostic gold standard will continue to be the rheumatologist's expert opinion (as we used in our study), not classification criteria.

Although we agree that our patients had lower severity scores on the widespread pain index (WPI) and polysymptomatic distress (PSD) than those in other studies, the mean WPI and PSD scores in our population still fell within the FM diagnostic threshold of the ACR criteria (WPI: 6.9 and PSD: 13.2). According to the study by Wolfe et al (4), the major approximate diagnostic separation lines start at a WPI score of 7 and a PSD score of 12. It must be highlighted that separation of patients according to these scales is arbitrary and represents a logically false classification. FM is a dynamic condition with a fluctuating course over time (5). One patient with FM with subsyndromal scores who does not satisfy the ACR criteria may reach the FM criteria threshold at another time. Furthermore, one question arises: What is the difference between persons with PSD scores of 10 and those with PSD scores of 12? It has been shown that persons who do not meet FM criteria but have mild (scores 4‐7) and moderate (scores 8‐11) PSD scores have substantial symptomatic burdens and an increase in adverse outcomes (6). Therefore, it appears that diagnosing this condition at the lower severity score is not a weak point of our study, but rather, it indicates that we should not delimit FM diagnosis to scores used in the existing criteria.

Although the ACR criteria have had a fundamental role in the definition and understanding of FM nature and provide important insights into the FM continuum concept, they depend on symptom‐based scoring and are prone to high subjectivity. NFF as a mechanistic‐based tool focuses on the attributes of nociplastic pain instead of counting symptoms and pain locations. It contains both FM core symptoms and pain attributes, such as migratory pattern, lack of response to analgesia, affective and social aspects, emotional and physical stressors, and hyperalgesia represented as modified tender points. Using NFF, physicians would be able to prospect beyond considering only the FM symptoms and shift their attention toward the essential features of nociplastic pain for early detection of this condition. In addition, these features appear to be less influenced by symptom fluctuations and severity because they are the embedded components of nociplastic pain. Thus, NFF differs from the previously described criteria in terms of its variables’ nature and independency to pain and/or symptoms extent.

At the same time, NFF or any mechanistic‐based approach still has a long way to go. As Dr. Wolfe pointed out, NFF must be examined in larger populations and different groups or geographic situations to confirm its validity and performance. We look forward to using and examining NFF in future research settings. However, acknowledgment of this new viewpoint not only does not seem to be a matter of concern but also can provide an opportunity for the nociplastic‐based approach for FM diagnosis.

## Supporting information


Disclosureform
